# Supplementary material for: Depletion of Abundant Sequences by Hybridization (DASH): using Cas9 to remove unwanted high-abundance species in sequencing libraries and molecular counting applications
Source: Genome Biol. 2016 Mar 4;17:41. doi: 10.1186/s13059-016-0904-5 (PMC4778327; doi:10.1186/s13059-016-0904-5)
Supplement: Additional file 1: Table S1. — List of all sgRNA sequences used in this paper. (PDF 34 kb) [file 13059_2016_904_MOESM1_ESM.pdf]

Supplemental Table 1: sgRNA Target Sequences

Name	Target Sequence	Name	Target Sequence
mt-rRNA-1	ATTTTCAGTGTATTGCTTTG	mt-rRNA-29	GGAACAGCTCTTTGGACACT
mt-rRNA-2	ACATCACCCCATAAACAAAT	mt-rRNA-30	GGCTGCTTTTAGGCCTACTA
mt-rRNA-3	AGGGTGAACTCACTGGAACG	mt-rRNA-31	TTTGGGATTTTTTAGGTAGT
mt-rRNA-4	TCTAAATCACCACGATCAAA	mt-rRNA-32	GATTGGTCCAATTGGGTGTG
mt-rRNA-5	TTTCCCGTGGGGGTGTGGCT	mt-rRNA-33	ACTAACATTAGTTCTTCTAT
mt-rRNA-6	AAACTTTCGTTTATTGCTAA	mt-rRNA-34	TGATCTGACGCAGGCTTATG
mt-rRNA-7	AATCGTGTGACCGCGGTGGC	mt-rRNA-35	TGTTGGTTGATTGTAGATAT
mt-rRNA-8	ATCTAAAACACTCTTTACGC	mt-rRNA-36	CTTATGAGCATGCCTGTGTT
mt-rRNA-9	ACTGGAGTTTTTTACAACTC	mt-rRNA-37	GAAAGGTTAAAAAAAGTAAA
mt-rRNA-10	CACAAAATAGACTACGAAAG	mt-rRNA-38	GCAGGCGGTGCCTCTAATAC
mt-rRNA-11	GGGGTATCTAATCCCAGTTT	mt-rRNA-39	TTTGCACGGTTAGGGTACCG
mt-rRNA-12	GATTTAACTGTTGAGGTTTA	mt-rRNA-40	CCTCGTGGAGCCATTCATAC
mt-rRNA-13	GTCCTTTGAGTTTTAAGCTG	mt-rRNA-41	CACGGGCAGGTCAATTTCAC
mt-rRNA-14	ACAGAACAGGCTCCTCTAGA	mt-rRNA-42	TAATAAATTAAAGCTCCATA
mt-rRNA-15	TATATAGGCTGAGCAAGAGG	mt-rRNA-43	TTAGGACCTGTGGGTTTGTT
mt-rRNA-16	TCTTCAGCAAACCCTGATGA	mt-rRNA-44	TGCATTAAAAATTTCGGTTG
mt-rRNA-17	CCCATTTCTTGCCACCTCAT	mt-rRNA-45	AAGTCTTAGCATGTACTGCT
mt-rRNA-18	TCGACCCTTAAGTTTCATAA	mt-rRNA-46	TGTTCCGTTGGTCAAGTTAT
mt-rRNA-19	TGAAACTTAAGGGTCGAAGG	mt-rRNA-47	GTTGATATGGACTCTAGAAT
mt-rRNA-20	GTATACTTGAGGAGGGTGAC	mt-rRNA-48	TACGACCTCGATGTTGGATC
mt-rRNA-21	CTTTGTGTTAAGCTACACTC	mt-rRNA-49	GATGGTGCAGCCGCTATTAA
mt-rRNA-22	AAGGTTGTCTGGTAGTAAGG	mt-rRNA-50	GGTCTGAACTCAGATCACGT
mt-rRNA-23	CATTTACCCAAATAAAGTAT	mt-rRNA-51	TCTTGTCCTTTCGTACAGGG
mt-rRNA-24	AGTCCTTGCTATATTATGCT	mt-rRNA-52	TGAGATGATATCATTTACGG
mt-rRNA-25	TAACTAGAAATAACTTTGCA	mt-rRNA-53	CCCACACCCACCCAAGAACA
mt-rRNA-26	CACTATTTTGCTACATAGAC	mt-rRNA-54	ACTTAAAACTTTACAGTCAG
mt-rRNA-27	CTACCGAGCCTGGTGATAGC	KRAS WT	AAACTTGTGGTAGTTGGAGC
mt-rRNA-28	AGGGGATTTAGAGGGTTCTG	Non-human control	ACAAATATTTTAATACATGA
