# Supplementary material for: Depletion of Abundant Sequences by Hybridization (DASH): using Cas9 to remove unwanted high-abundance species in sequencing libraries and molecular counting applications
Source: Genome Biol. 2016 Mar 4;17:41. doi: 10.1186/s13059-016-0904-5 (PMC4778327; doi:10.1186/s13059-016-0904-5)
Supplement: Additional file 2: Figure S1. — Depletion efficiency by Cas9 dosage. (PDF 10688 kb) [file 13059_2016_904_MOESM2_ESM.pdf]

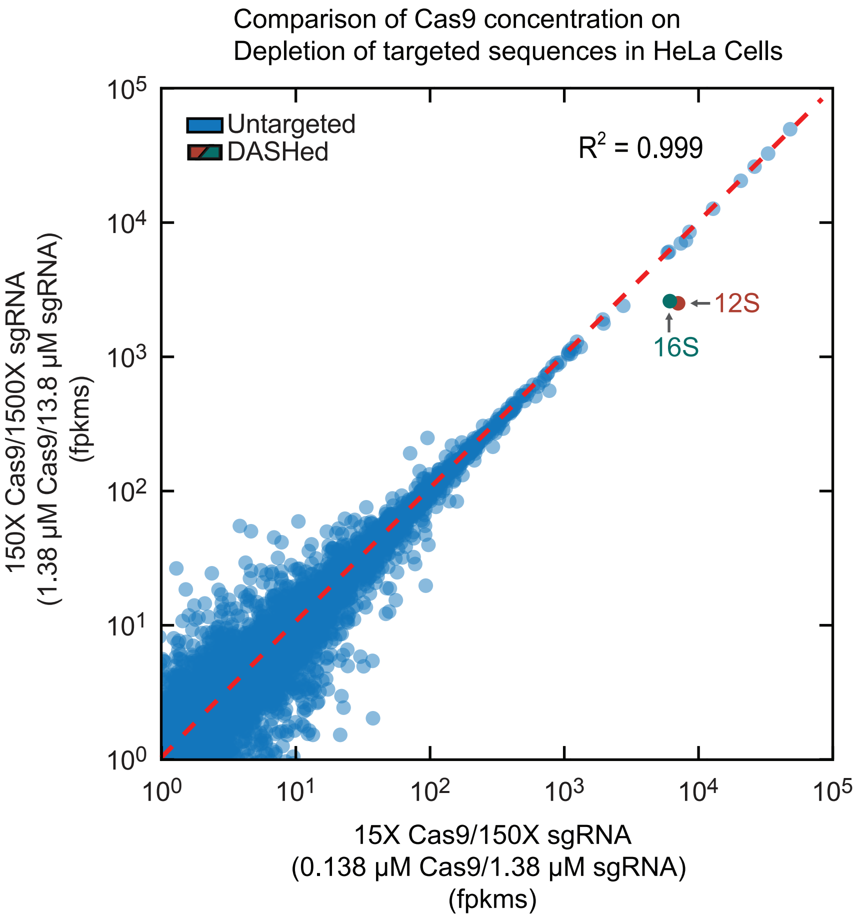

Supplemental Figure 1: Scatterplot of log of fragments per kilobase of transcript per million mapped reads (log-fpkm) values per human gene comparing HeLa cells DASHed with two different Cas9/sgRNA concentrations.
