# Supplementary material for: Depletion of Abundant Sequences by Hybridization (DASH): using Cas9 to remove unwanted high-abundance species in sequencing libraries and molecular counting applications
Source: Genome Biol. 2016 Mar 4;17:41. doi: 10.1186/s13059-016-0904-5 (PMC4778327; doi:10.1186/s13059-016-0904-5)
Supplement: Additional file 3: Figure S2. — PAM sites in a pseudogene. (PDF 442 kb) [file 13059_2016_904_MOESM3_ESM.pdf]

```
mt-rRNA 16S   AAAGTGCACTTGGACGAACCAGAGTGTAGCTTAACACAAAGCACCCAACTTACACTTAGGAGATTTCAACTTAACTTGACCGCTCTGAGCTAAACCTAGC 100
mtRNR2L 12    AAAGTGCACTTGGACGAACCAGAGTGTAGCTTAACATAAAGCACCCAACTTACACTTAGGAGATTTCAACTCAACTTGACCACTCTGAGCCAAACCTAGC 100

mt-rRNA 16S   CCCAAACCCACTCCACCTTACTACCAGACAACCTTAGCCAAACCATTTACCCAAATAAAGTATAGGCGATAGAAATTGAAACCTGGCGCAATAGATATAG 200
mtRNR2L 12    CCTAAACCCGTTCCACCTTACTATCAAATAACCTTAACCAAACCATTTACCCAAATAAAGTATAGGCGATAGAAATTGTAAACCGGCGCAATAGATATAG 200

mt-rRNA 16S   TACCGCAAGGGAAAGATGAAAAATTATAACCAAGCATAATATAGCAAGGACTAACCCCTATACCTTCTGCATAATGAATTAACTAGAAATAACTTTGCAA 300
mtRNR2L 12    TACCGCAAGGGAAAGATGAAAAATTATAACCAAGCATAATACAGCAAGGACTAACCCCTGTACCTTTTGCATAATGAATTAACTAGAAATAACTTTGCAA 300

mt-rRNA 16S   GGAGAGCCAAAGCTAAGACCCCCGAAACCAGACGAGCTACCTAAGAACAGCTAAAAGAGCACACCCGTCTATGTAGCAAAATAGTGGGAAGATTTATAGG 400
mtRNR2L 12    AGAGAACCAAAGCTAAGGCCCCCGAAACCAGACGAGCTACCTAAGAACAGCTAAAAGAGCACACCCGTCTATGTAGCAAAATAGTGGGAAGATTTATAGG 400

mt-rRNA 16S   TAGAGGCGACAAACCTACCGAGCCTGGTGATAGCTGGTTGTCCAAGATAGAATCTTAGTTCAACTTTAAATTTGCCCACAGAACCCTCTAAATCCCCTTG 500
mtRNR2L 12    TAGAGGCGACAAACCTATCGAGCCTGGTGATAGCTGGTTGTCCAAGATAGAATCTTAGTTCAACTTTAAATTTACCTACAGAACCTTCTAAATCCCCTTG 500

mt-rRNA 16S   TAAATTTAACTGTTAGTCCAAAGAGGAACAGCTCTTTGGACACTAGGAAAAAACCTTGTAGAGAGAGTAAAAAATTTAACACCCATAGTAGGCCTAAAAG 600
mtRNR2L 12    TAAATTTAACTGTTAGTCCAAAGAGGAACAGCTCTTTGGACACTAGGAAAAAACCTTGTAAAGAGAGTAAAAAATTTAATACCCATAGTAGGCCTAAAAG 600

mt-rRNA 16S   CAGCCACCAATTAAGAAAGCGTTCAAGCTCAACACCCACTACCTAAAAAATCCCAAACATATAACTGAACTCCTCACACCCAATTGGACCAATCTATCAC 700
mtRNR2L 12    CAGCCACCAATTAAGAAAGCGTTCAAGCTCAACACCCATCGTCTAAAAAATCCCAAACATACAACTGAGCTCCTTACACTCAATTGGACCAATCTATTAC 700

mt-rRNA 16S   CCTATAGAAGAACTAATGTTAGTATAAGTAACATGAAAACATTCTCCTCCGCATAAGCCTGCGTCAGATCAAAACACTGAACTGACAATTAACAGCCCAA 800
mtRNR2L 12    CTTATAGAAGAACTAATGTTAGTATAAGTAACATGAAAACATTCTCCTCCGCATAAGCCTACATCAGACCAAAATATTAAACTGACAATTAACAGCCCAA 800

mt-rRNA 16S   TATCTACAATCAACCAACAAGTCATTATTACCCTCACTGTCAACCCAACACAGGCATGCTCATAAGGAAAGGTTAAAAAAAGTAAAAGGAACTCGGCAAA 900
mtRNR2L 12    TATCTACAATCAACCAACAAGCCATTATTACCCTCACTGTTAACCCAACACAGGCATGCCCACAAGGAAAGGTTAAAAAAAGTAAAAGGAACTCGGCAAA 900

mt-rRNA 16S   CCTTACCCCGCCTGTTTACCAAAAACATCACCTCTAGCATCACCAGTATTAGAGGCACCGCCTGCCCAGTGACACATGTTTAACGGCCGCGGTACCCTAA 1000
mtRNR2L 12    TCTTACCCCGCCTGTTTACCAAAAACATCACCTCTAGCATTATCAGTATTAGAGGCACCGCCTGCCCGGTGACATATGTTTAACGGCCGCGGTACCCTAA 1000

mt-rRNA 16S   CCGTGCAAAGGTAGCATAATCACTTGTTCCTTAAATAGGGACCTGTATGAATGGCTCCACGAGGGTTCAGCTGTCTCTTACTTTTAACCAGTGAAATTGA 1100
mtRNR2L 12    CCGTGCAAAGGTAGCATAATCACTTGTTCCTTAAATAGGGACTTGTATGAATGGCTCCACGAGGGTTCAGCTGTCTCTTACTTTCAACCAGTGAAATTGA 1100

mt-rRNA 16S   CCTGCCCGTGAAGAGGCGGGCATGACACAGCAAGACGAGAAGACCCTATGGAGCTTTAATTTATTAATGCAAACAGTACCTAACAAACCCACAGGTCCTA 1200
mtRNR2L 12    CCTACCCGTGAAGAGGCGGGCATAACATAGCAAGACTATGTTACTACATACAACTAACATGTTACTGAGCAAGTTTTTCTCAAAAAAGTAGAATGCTAGG 1200

mt-rRNA 16S   AACTACCAAACCTGCATTAAAAATTTCGGTTGGGGCGAC
mtRNR2L 12    ATTATAGAAAAATA-ATTAAAATAATACATTTCACCCTT
```

Key to mtRNR2L 12 sequence:
- PAM
- No PAM
- match
- mismatch
- sgRNA target, perfect match
- sgRNA target, some mismatches

Supplemental Figure 2: mt rRNA sgRNA target sites in the pseudogene mtRNR2L 12.
