# Supplementary material for: Depletion of Abundant Sequences by Hybridization (DASH): using Cas9 to remove unwanted high-abundance species in sequencing libraries and molecular counting applications
Source: Genome Biol. 2016 Mar 4;17:41. doi: 10.1186/s13059-016-0904-5 (PMC4778327; doi:10.1186/s13059-016-0904-5)
Supplement: Additional file 4: Figure S3. — Scatter plots for patient samples. (PDF 8093 kb) [file 13059_2016_904_MOESM4_ESM.pdf]

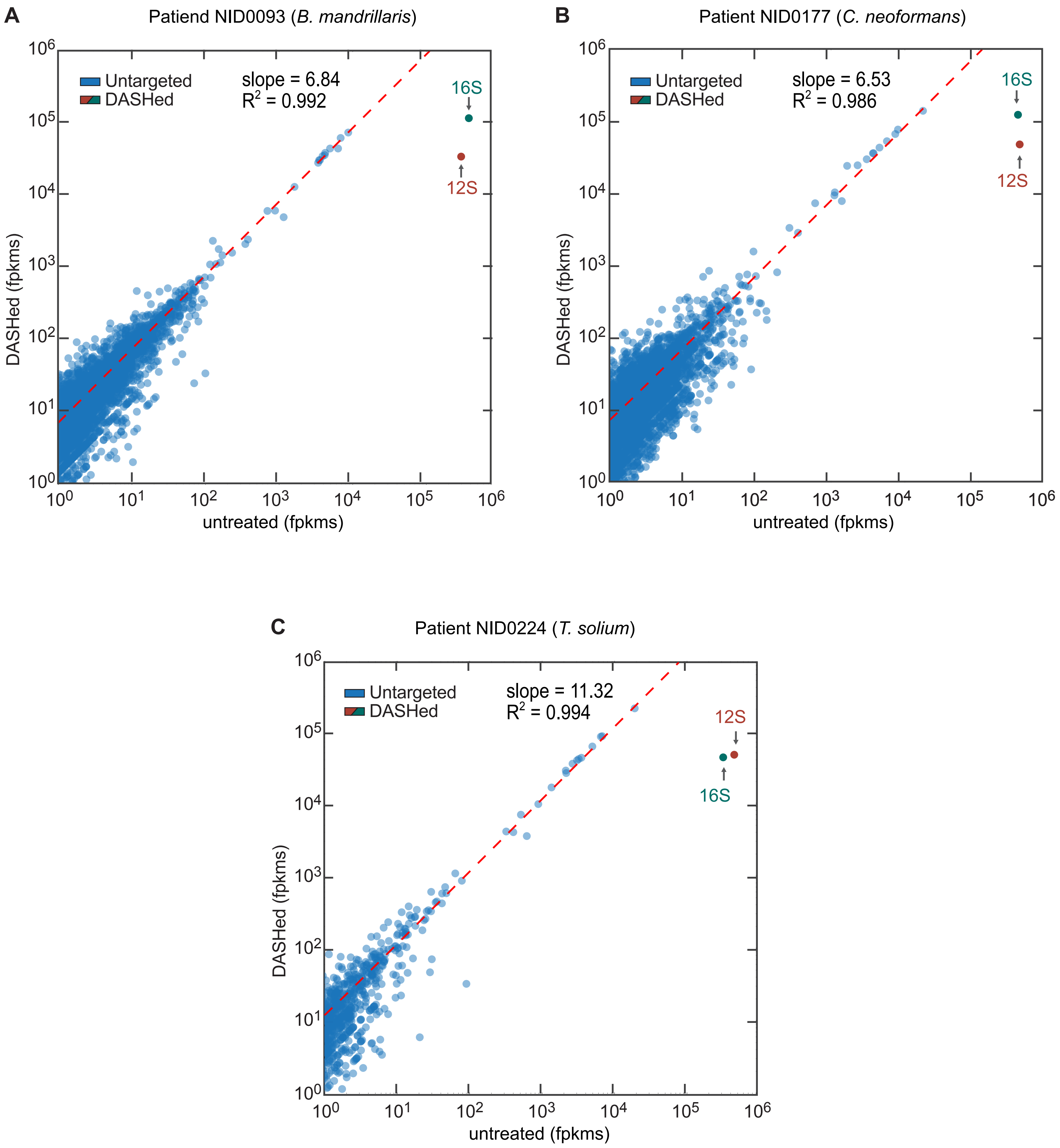

Supplemental Figure 3: Scatterplots of the log of fragments per kilobase of transcript per million mapped reads (log-fpkm) values per human gene in the DASHed vs. untreated patient samples. The slopes of the regression lines (red) indicate the fold enrichment in reads mapped to untargeted transcripts. R-squared (R^2) values of the regression lines indicate minimal off-target depletion.
